# Population Preference of Net Texture prior to Bed Net Trial in Kala-Azar–Endemic Areas

**DOI:** 10.1371/journal.pntd.0000100

**Published:** 2007-12-12

**Authors:** Murari L. Das, Shri P. Singh, Veerle Vanlerberghe, Suman Rijal, Madhukar Rai, Prahlad Karki, Shyam Sundar, Marleen Boelaert

**Affiliations:** 1 BP Koirala Institute of Health Sciences, Dharan, Nepal; 2 Institute of Medical Sciences, Banaras Hindu University, Varanasi, India; 3 Institute of Tropical Medicine, Antwerp, Belgium; George Washington University, United States of America

## Abstract

Prior to a community-based efficacy trial of long-lasting insecticidal nets (LLINs) in the prevention of visceral leishmaniasis (VL; also called kala-azar), a pilot study on preference of tools was held in endemic areas of India and Nepal in September 2005.

LLINs made of polyester and polyethylene were distributed to 60 participants, who used the nets sequentially for 7 d. Acceptability and preference were evaluated via indirect indicators through questionnaires at three defined time points before and after use of the LLINs and through focus group discussions (FGDs). In the latter, preferences for color and size were also assessed. Untreated bed nets were owned by 87% of the households prior to the study. All users liked textures of both LLIN types after 7 d of use, but had a slight preference for those made of polyester if they were to recommend a LLIN to relatives or friends (*p*<0.05), mainly because of their relatively greater softness in comparison to polyethylene LLINs. Users reported that both net types reduced mosquito bites and number of insects, including sand fly (bhusana; genus *Phlebotomus*), inside the house. Side effects were minor and disappeared quickly.

The large-scale intervention trial considered the preferences of the study population to decide on the best tool of intervention—light-blue, rectangular, polyester LLINs of different sizes.

## Author Summary

Visceral leishmaniasis (VL) is a neglected, life-threatening, vector-borne disease. More than 90% of the reported VL cases occur in the Sudan and the Indian subcontinent, where it is considered a problem of great public health importance. To improve its control, which is currently mainly based on case detection and treatment, research is needed on preventive measures, such as the use of bed nets impregnated with long-lasting insecticide (LLINs). Prior to an efficacy trial on LLINs, we conducted a pilot study to assess community preferences for size, color, and texture of bed nets. Such an acceptance study aims at evaluating user preferences as a way to maximize usage and, consequently, to anticipate how effective a control tool might become. That pilot study concluded that different textures and colors of LLIN are accepted by users, although there was a slight preference for the softer polyester net. These results were utilized in the large-scale efficacy trial in order to maximize the coverage, uptake, and use of the LLINs.

## Introduction

Annually 500,000 new cases of visceral leishmaniasis (VL, also called kala-azar), with 59,000 deaths, are reported in 62 countries. More than 90% of the cases occur in the Indian subcontinent and Sudan [1]. In India and Nepal, VL is caused by *Leishmania donovani*, and the sand fly, *Phlebotomus argentipes*, is the only proven vector [2]. Insecticide-treated nets (ITNs) have shown their effectiveness in the prevention of malaria and cutaneous leishmaniasis [3]–[6]. ITNs have also been effective in reducing VL vectors in an endemic region of Brazil [7]. Two studies have suggested that VL incidence may be reduced when ITNs are used [8],[9], but no randomized controlled trial has clearly demonstrated the impact of ITNs on VL. To evaluate the efficacy of these tools on VL transmission, a large-scale randomized controlled community intervention trial implementing long-lasting insecticidal nets (LLINs) was planned in VL-endemic areas of India and Nepal. LLINs are pretreated bed nets that do not need retreatment after washing and have insecticidal activity lasting for a number of years. Hence, this pilot study was undertaken with the objective of assessing community preferences for size, color, and brand (texture) of net, which information was used in launching the large-scale community efficacy trial.

## Materials and Methods

The study was carried out in two rural VL-endemic villages, Phanda (Muzaffarpur district, India) and Sidraha (Morang district, Nepal), randomly chosen in the region where the bed net trial would be implemented. In total 60 houses were identified in the main streets of the villages in September 2005.

All included households were approached individually, and procedures and objective of the study were explained to household members. One person per household (in total 60 persons), irrespective of age and sex, and capable of assessing advantages and disadvantages of nets, were selected based on their willingness to participate in the survey and to use a LLIN for 14 nights.

Light-blue polyethylene and polyester bed nets were distributed to users. The polyethylene nets are treated with 1,000 mg/m^2^ permethrin, have wide mesh (4×4 mm), and have fiber thickness of 150 denier (Olyset, Sumitomo Chemical Company, Japan). The polyester nets have a resin coating containing 55 mg/m^2^ Deltamethrin, small mesh (1.5×1.5 mm), and fiber thickness of 100 denier (PermaNet 2.0, Vestergaard-Frandsen, Denmark). The two brands of LLIN are currently approved by World Health Organization Pesticide Evaluation Scheme (WHOPES) and are widely commercially available [10].

Over the 15 d trial period, 60 rectangular nets (30 in Nepal and 30 in India) of each material were distributed, 50 double-sized (130×180×150 cm) and ten family-sized (160×180×150 cm). Family-sized nets were given to participants who slept in groups of more than three persons. In India all 30 households used the polyethylene nets during the first 7 d. On day 8 these nets were withdrawn and polyester nets were distributed to the same participants and used for 7 d. In Nepal a crossover design was implemented. Fifteen participants used polyethylene nets for the first 7 d, while another 15 used polyester nets. On day 8 all nets were removed and new nets of the other material were given, to be used until day 15.

Preference and acceptability, which are subjective measures based on attitudes of the participant, were evaluated through questionnaires and focus group discussions (FGDs). Pretested, structured questionnaires in the appropriate national language were administered to the respondents on day 0 (predistribution survey), day 8 (first round), and day 15 (second round). In the predistribution survey, ownership and use of traditional bed nets were explored. On days 8 and 15 the perception of the user of the LLIN used over the previous 7 d was evaluated. In the second-round survey an additional question was inserted on the preference for type of net.

The quantitative data were entered in an Excel data sheet, proportions of variables were calculated, and the difference between groups was evaluated for significance by the Chi-square test. On day 15, four 1-h focus groups were conducted, each with seven or eight LLIN users in the presence of two moderators. Each user's perception of each type of LLIN was explored, as was his or her preference for one net material, color, and size. Respondents were shown the two above-mentioned sizes and different colors of the two brands (white and blue polyethylene nets; khaki, pink, yellow, dark blue, light brown, and Madagascar green polyester nets). The preference of the users for texture, color, and size were assessed through a content analysis of the recordings of each FGD.

Ethical clearance for the protocol and informed consent forms were obtained from the institutional review boards of B. P. Koirala Institute of Health Sciences, Dharan, Nepal and the Institute of Medical Sciences, Banaras Hindu University, India. Written and/or oral informed consent was obtained from all study participants and their households.

## Results

Prior to the study, 87% of the 60 households (80% in India, 93% in Nepal) had on average 2.6 mosquito nets per house (range 1–6). These bed nets were not insecticide-impregnated, were mostly double sized, and made of nylon. Only 62% were clean and 73% were hanging correctly, and less than half were intact (46%). The colors of nets owned varied: 25% green, 25% pink or red, 17% blue, 13% yellow, 11% white, and 9% khaki. 83% of the respondents used mosquito nets to be protected from mosquito bites. Close to half of the respondents mentioned also its protective character from other insects and animals. Of the households owning bed nets, only 54% used them the whole year through, the other half used them mainly during the seasons with high mosquito nuisance levels. In the summer season, some households avoided using bed nets because of the high temperatures.

Females constituted 52% of the respondents. The age groups 15–30 y, 31–40 y, 41–50 y, and above 51 y respectively constituted 38%, 28%, 17%, and 17% of the participants.

### Use of LLINs

In the majority of cases, only one or two persons slept under one LLIN, but in one case five persons slept under one LLIN. Nets were not used for two and one nights, respectively, by 5% and 3% of polyester users and 2% and 10% of polyethylene users. Different reasons for not using the LLIN mentioned by respondents were leaking of rain water from roof, not finding the material to tie the net, used by parent or guest, or user spent the night out of the village. 72% of the LLINs were used indoors. In Nepal, 97% hung the LLIN with rope on the wall, in India 63% placed their LLIN on sticks. During the day, in India 70% of the households kept the LLINs folded and aside, whereas in Nepal the majority (66%) kept the net hanging on the bed with the sides raised. All nets were hung correctly and kept clean and intact.

### Preference of Brand and Perception of Side Effects

All users claimed to accept both nets. The advantages of polyethylene LLINs observed by the users were the following: 73% of the respondents indicated a reduction of mosquito bites, 28% a reduction of insects in the house, and 17% a sense of pleasant sleep under the net, versus, for the polyester LLINs, respectively 85% (*p* = 0.11), 50% (*p* = 0.01), and 65% (*p*<0.01). On days 7 and 15, after 7 d use of polyester and polyethylene LLINs, itching was reported by 18% and 33% (*p* = 0.06) of respondents, respectively, and sneezing was reported by 12% and 22% (*p* = 0.14), respectively. These findings were confirmed by the users during the FGD on day 15, after both types of nets were used. These adverse effects were minor and were noticed for the first few days of use of the respective nets.

**Table 1:** User Preferences for Two Types of LLIN in Endemic Areas of Visceral Leishmaniasis

Category	Subcategory	India		Nepal		Total	
		Polyester, *n* (%)	Polyethylene, *n* (%)	Polyester, *n* (%)	Polyethylene, *n* (%)	Polyester, *n* (%)	Polyethylene, *n* (%)
Advantages of using LLINs^a^	Reduction in mosquito bites	24 (80)	24 (80)	27 (90)	20 (67)	51 (85)	44 (73)
	Reduction of insects inside the house	5 (17)	3 (10)	25 (83)	14 (47)	30 (50)	17 (28)*
	Pleasant sleep at night	18 (60)	2 (7)	21 (70)	8 (27)	39 (65)	10 (17)*
Adverse effects	Itching^b^	9 (30)	9 (30)	2 (7)	11 (37)	11 (18)	20 (33)
	Sneezing^b^	5 (16)	6 (20)	2 (7)	7 (23)	7 (12)	13 (22)
Willing to use the LLIN in the future (by users)		30 (100)	27 (90)	30 (100)	21 (70)	60 (100)	48 (80)*
Recommend the use of LLIN to non-using persons		30 (100)	27 (90)	30 (100)	22 (73)	60 (100)	49 (82)*

*: Differences are significant at *p*<0.05.

a Due to multiple responses, the percentages exceed 100.

b Reports of itching and sneezing increased after the first interview.

But when participants were asked which type they would recommend to their family members and friends, 100% of the study population indicated polyester nets, versus 82% for polyethylene (*p* = 0.001). Various reasons were given for this preference for polyester nets: 50% of the users preferred the combination of reduced insect (including sandflies, or bhusana) bites together with the softness of the polyester net; 43% mentioned reduced insect bites as the only reason; and 7% mentioned the combination of reduced insect bites together with a feeling of pleasant sleep. The reason 82% recommended polyethylene nets were, in all cases, because of the reduced number of insect bites; 7% added that aeration was good when sleeping under this type of net. The 11 users that would not recommend the polyethylene nets gave as reasons the roughness of the net (nine users) and large mesh size of this type of net, which permits the entry of mosquitoes (two users).

During the FGD it was stated by the users that in comparison with polyester nets, the users disliked polyethylene nets primarily because of their rough texture, and secondarily for their side effects.

### Preference for Color and Size

The qualitative and quantitative data showed that more than one-third of the users preferred the light blue color, followed by green and khaki. A difference between male and female responders was observed: females preferred blue, before green and khaki; male preferred green, before khaki and blue. The male respondents disliked the color of currently used nets. Their experience was that mosquitoes were attracted to the dark colors of the forest and therefore expressed a preference for lighter colors. All were satisfied with the size of the net provided except one respondent in Nepal living in a house with large sleeping groups (five persons sleeping under one net). Hence a family-size net was not large enough for that sleeping group.

## Discussion

We can conclude from this pilot study that both brands of LLIN had high acceptability in the VL-endemic areas of Nepal and India. A high percentage of households in these areas possess bed nets, which has also been described in other studies [11],[12]. In the study group, none of the existing nets were insecticide-impregnated, and participants were willing to trade their nets for any of the LLINs. Regardless of the study design used, the same trend in Indian and Nepalese data can be observed (as presented in Table 1), i.e., polyester LLINs (because of relative softness and pleasant sleep experience) were preferred to polyethylene. The discrepancies between the color of nets actually owned by the households and their preferences is due to the nonavailability of these colors on the local market. A high percentage of users reported side effects with the LLINs in the first few days of use of each type, but these were minor and disappeared quickly. More side effects were reported with polyethylene nets than with polyester, but this difference was not significant. Because of raised awareness of the respondents, the reporting of side effects was higher in the second round of the study compared to the first.

Because this study was conducted in preparation for a large-scale efficacy trial, it was small in scale and we recognize its limitations. First, the period of observation was short, and persons were included in the study based on their willingness to use a LLIN for 2 wk. The majority (87%) of participants belonged to households that already had untreated mosquito nets in their homes prior to this pilot study. Therefore, this study documented participants' preference to change from untreated nets to one or another brand of LLIN. Another limitation is the nonrandom assignment of types of net to use, which can cause an information bias, as the second type of net used was intuitively compared to the first one. We addressed this problem by taking the questionnaire on day 7 before switching the nets and by the crossover design in the Nepalese part of the study. Third, this preliminary indication of acceptability might not necessarily translate into use, as was shown by Jima et al. [13] in Ethiopia, who found that although acceptance of and willingness to use ITNs for malaria prevention was very high, actual utilization of the mosquito nets was very low because of lack of knowledge, unavailability of nets, and low household purchase power. LLINs show promise as a tool for controlling VL, since the density of VL vectors peaks between 20:00 and 24:00 hours [7],[14].

The preference of the population to use light-blue, rectangular polyester LLINs was used in choosing the tool to implement in the large-scale trial. The problems concerning the use of LLINs identified by the population guided the researchers during the development of the promotional and educational messages that accompanied the implementation.
